# Overexpression of miR-17 predicts adverse prognosis and disease recurrence for acute myeloid leukemia

**DOI:** 10.1007/s10147-022-02161-5

**Published:** 2022-05-10

**Authors:** Yang Cao, Yue Liu, Limei Shang, Huijuan Chen, Yanhua Yue, Weimin Dong, Yanting Guo, Haonan Yang, Xiaojun Yang, Yan Liu, Weiying Gu, Xiaoying Zhang

**Affiliations:** 1 Department of Hematology, The First People’s Hospital of Changzhou, The Third Affiliated Hospital of Soochow University, Changzhou, 213003 Jiangsu China; 2 Department of Blood Transfusion, The First People’s Hospital of Changzhou, The Third Affiliated Hospital of Soochow University, Changzhou, 213003 Jiangsu China; 3 Comprehensive Laboratory, The First People’s Hospital of Changzhou, The Third Affiliated Hospital of Soochow University, Changzhou, 213003 Jiangsu China

**Keywords:** miR-17, Expression, Acute myeloid leukemia, Prognosis, Recurrence

## Abstract

**Background:**

The clinical significance of miR-17 in patients with acute myeloid leukemia (AML) remains unknown.

**Methods:**

Real-time quantitative reverse transcription-polymerase chain reaction (qPCR) was performed to detect the miR-17 expression in 115 de novo AML patients, 31 patients at complete remission (CR) time, 8 patients at relapse time and 30 normal controls.

**Results:**

MiR-17 was upregulated in de novo AML compared with normal controls. Patients with high expression of miR-17 had less CEBPA double mutation, less favorable ELN-risk and lower CR rate. The level of miR-17 was significantly decreased at CR phase and was returned to primary level even higher when in relapse phase. In addition, Cox regression analysis revealed that miR-17 expression retained independent prognostic significance for overall survival (OS). Moreover, the gene-expression profile analysis of miR-17 in AML obtained from TCGA database was involved in multiple biological functions and signal pathways. Among the differential expressed genes (DEGs), we identified FGL2, PLAUR, SLC2A3, GPR65, CTSS, TLR7, S1PR3, OGFRL1, LILRB1, IL17RA, SIGLEC10, SLAMF7, PLXDC2, HPSE, TCF7 and MYCL as potential direct targets of miR-17 according to in silico analysis.

**Conclusions:**

High expression of miR-17 in de novo AML patients pointed to dismal clinical outcome and disease recurrence, which could serve as novel prognostic biomarker for AML patients.

## Introduction

As the most common type of leukemia in adults, acute myeloid leukemia (AML) is a malignant clone disease of the hematopoietic system. Although there have been great advances in the treatment of AML, patients have highly heterogenous clinical course and their long-term prognosis is still dismal [1]. Therefore, identifying novel prognostic markers to improve the existing molecular-based stratification and risk-adapted therapy for AML patients is urgently required.

It is well known that microRNAs (miRNAs) have been implicated in both biological and pathological processes such as cell growth, apoptosis, migration and invasion. There is an increasing body of evidence unraveling the molecular mechanism of miRNAs as either oncogenes or tumor suppressors in human cancers, which is mostly depending on the function of their different target genes and specific tumor microenvironment [2]. Aberrantly expressed miRNA patterns with clinical prognostic significance have been reported in AML patients [3]. For instance, in a cohort of 176 AML patients from TCGA database, the data of miRNAs sequencing and gene microarray were analyzed to identify risk miRNAs with prognostic value. Among the 705 miRNAs that were studied, upregulated miR-520a, 599, 606, 137 and 362 predicted unfavorable outcomes of AML patients [4]. In another study of 187 cytogenetically normal AML (CN-AML) patients, miR-181a overexpression was strongly correlated with better survival [5].

The miR-17-92 cluster, known as OncomiR-1, is a highly conserved polycistronic transcript among vertebrates, which is located on the open reading frame 25 of chromosome 13 (C13orf25) and yields six mature miRNAs (miR-17, 18a, 19a, 20a, 19b, and 92a) [6]. Accumulating evidence have revealed that the members of miR-17-92 family are very often dysregulated and play critical roles in a wide range of cancer types including osteosarcoma, hepatocellular carcinoma, renal cancer, ovarian cancer, lymphoma, retinoblastoma and so on [7]. Whereas, the expression and prognostic significance of miR-17 in AML has not been fully investigated. In current study, we aimed to evaluate the expression and clinical significance of miR-17 in de novo AML patients.

## Materials and methods

### Patients and samples

In accordance with the Declaration of Helsinki Principles, written informed consents were obtained from all patients. A total of 115 newly diagnosed AML patients and 30 healthy controls were enrolled in this study. Bone marrow (BM) samples were collected from the 30 healthy controls, 115 AML patients at primary diagnosis, 31 patients at complete remission (CR), and 8 patients at relapse.

### RNA extraction, reverse transcription and real-time quantitative PCR

Briefly, RNA was extracted from Ficoll-separated BM mononuclear cells with Trizol (Thermo Fisher Scientific, USA) following the protocol of the manufacture. MiR-17 expression was determined using the TaqMan MicroRNA Assay (Thermo Fisher Scientific, USA). Reverse transcription was performed to synthesize cDNA using the TaqMan MicroRNA Reverse Transcription kit (Thermo Fisher Scientific, USA). The quantification of miR-17 expression was conducted using the TaqMan PCR Master Mix (Assay ID: 000,393, Thermo Fisher Scientific, USA). RNU6B were selected as endogenous controls. Relative miR-17 expression level was calculated by 2^−△△CT^ method. All experiments were run in triplicate.

### Statistical analysis

Pearson Chi-square analysis and Fisher’s exact test were carried to compared the differences of categorical variables between two groups, while Mann–Whitney *U* test and *T* test were carried to compared the differences of continuous variables between two groups. Wilcoxon singed rank test were conducted to compared the differences of matched patients. The effect of miR-17 expression on clinical outcome were evaluated by Kaplan–Meier and Cox regression model. *P* < 0.05 was considered as statistically significant. SPSS 25.0 software was conducted for statistical analyzes.

## Results

### The upregulated miR‐17 expression in BM of AML patients

**Fig. 1:**
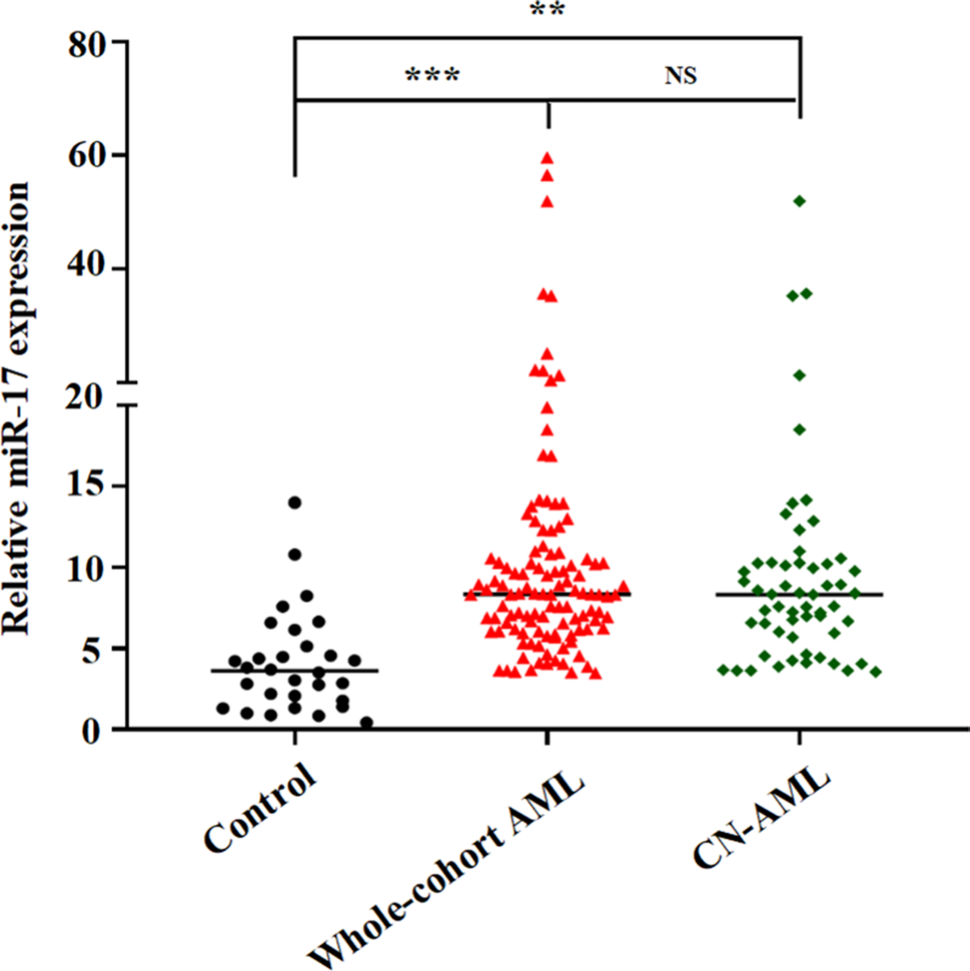
BM miR-17 expression in controls and AML patients. The distributions of miR‐17 expression in controls, whole-cohort AML and CN‐AML were presented with scatter plots. The median level of miR‐17 expression in each group was shown with horizontal line. *AML* acute myeloid leukemia; *CN‐AML* cytogenetically normal AML. **P < 0.01; ***P < 0.001; ^NS^*P* > 0.05. Data are shown as mean from three independent experiments

We detected the expression of miR-17 in BM from 30 healthy donors and 115 AML patient (including 57 CN-AML patients). As shown in Fig. 1, the relative level of miR-17 in AML patients ranged from 3.49 to 59.62 (median 8.34), which was significantly higher than that in normal controls (median 3.62, range 0.42–14.01, *P* < 0.0001). In addition, significant overexpression of BM miR-17 expression was also presented in CN-AML patients.

### Correlation of BM miR-17 level with clinical features in AML

To analyze the clinical relevance of miR-17 expression with AML, the whole-cohort patients were divided into high and low miR-17 expression groups based on the its median level. The association of the clinical and genetic characteristics with miR-17 expression was shown in Table 1. The low miR-17 expression group had more patients with CEBPA double mutation (*P* = 0.029) and favorable ELN-risk (*P* = 0.047) than the high miR-17 group. There were no strikingly differences in sex, age, white blood cells, hemoglobin, platelets, BM blasts, FAB subtypes and common fusion genes between the high and low miR-17 expression groups.

**Table 1:** Correlation of miR-17 expression with clinical/laboratory features in AML patients

Patient's features	Total (n = 115)	High (n = 57)	Low (n = 58)	*P* value
Sex, male/female	62/53	30/27	32/26	0.785
Median age, years (range)	57 (18–88)	58 (19–88)	55.5 (18–82)	0.538
≥ 60/ < 60, years	55/60	27/30	28/30	0.922
Median WBC, × 10^9^/L (range)	11.4 (0.4–470.3)	12.9 (0.4–295.5)	10.9 (1.0–470.3)	0.765
≥ 20/ < 20, 10^9^/L	48/67	26/31	22/36	0.404
Mean Hb, g/L ± SD	83.3 ± 23.8	79.1 ± 21.5	87.4 ± 25.4	0.061
Median PLT, × 10^9^/L (range)	43 (5–946)	45 (6–946)	37.5 (5–832)	0.437
Median BM blasts, % (range)	66 (21–97)	66 (21–97)	67.25 (22–94)	0.900
≥ 50/ < 50, %	85/30	44/13	41/17	0.427
FAB subtypes, n (%)				
M0	1 (0.9)	0 (0.0)	1 (1.7)	1.000
M1	20 (17.4)	6 (10.5)	14 (24.1)	0.054
M2	58 (50.4)	32 (56.1)	26 (44.8)	0.225
M4	2 (1.7)	1 (1.8)	1 (1.7)	1.000
M5	26 (22.6)	13 (22.8)	13 (22.4)	0.960
No data	8 (7.0)	5 (8.8)	3 (5.2)	0.695
ELN risk, n (%)				
Favorable	34 (29.6)	12 (21.1)	22 (37.9)	**0.047**
Intermediate	57 (49.6)	29 (50.9)	28 (48.3)	0.780
Adverse	24 (20.9)	16 (28.1)	8 (13.8)	0.053
Gene mutations, n (%)				
TP53 ( +)	8 (7.0)	6 (10.5)	2 (3.4)	0.261
CEBPA double ( +)	11 (9.6)	2 (3.5)	9 (15.5)	**0.029**
NPM1 ( +)	15 (13.0)	9 (15.8)	6 (10.3)	0.386
FLT3-ITD ( +)	15 (13.0)	6 (10.5)	9 (15.5)	0.427
MLL-PTD ( +)	2 (1.7)	2 (3.5)	0 (0.0)	0.243
TET2 ( +)	16 (13.9)	9 (15.8)	7 (12.1)	0.564
IDH1 ( +)	5 (4.3)	3 (5.3)	2 (3.4)	0.984
IDH2 ( +)	17 (14.8)	7 (12.3)	10 (17.2)	0.454
DNMT3A ( +)	19 (16.5)	12 (21.1)	7 (12.1)	0.195
ASXL1 ( +)	5 (4.3)	3 (5.3)	2 (3.4)	0.984
c-kit ( +)	9 (7.8)	5 (8.8)	4 (6.9)	0.978
RUNX1 ( +)	9 (7.8)	3 (5.3)	6 (10.3)	0.505
NRAS ( +)	13 (11.3)	8 (14.0)	5 (8.6)	0.359
KRAS ( +)	6 (5.2)	5 (8.8)	1 (1.7)	0.201
Fusion gene, n (%)				
AML-ETO	11 (9.6)	5 (8.8)	6 (10.3)	0.774
BCR-ABL	1 (0.9)	0 (0.0)	1 (1.7)	1.000
CBFβ-MYH11	8 (7.0)	4 (7.0)	4 (6.9)	1.000
MLL-related	2 (1.7)	2 (3.5)	0 (0.0)	0.243
CR ( ±)	71/44	27/30	44/14	**0.002**

*WBC* white blood cells, *Hb* haemoglobin, *PLT* platelet, *BM* bone marrow, *CR* complete remission

### Low expression of miR-17 is predictive for favorable survival

**Fig. 2:**
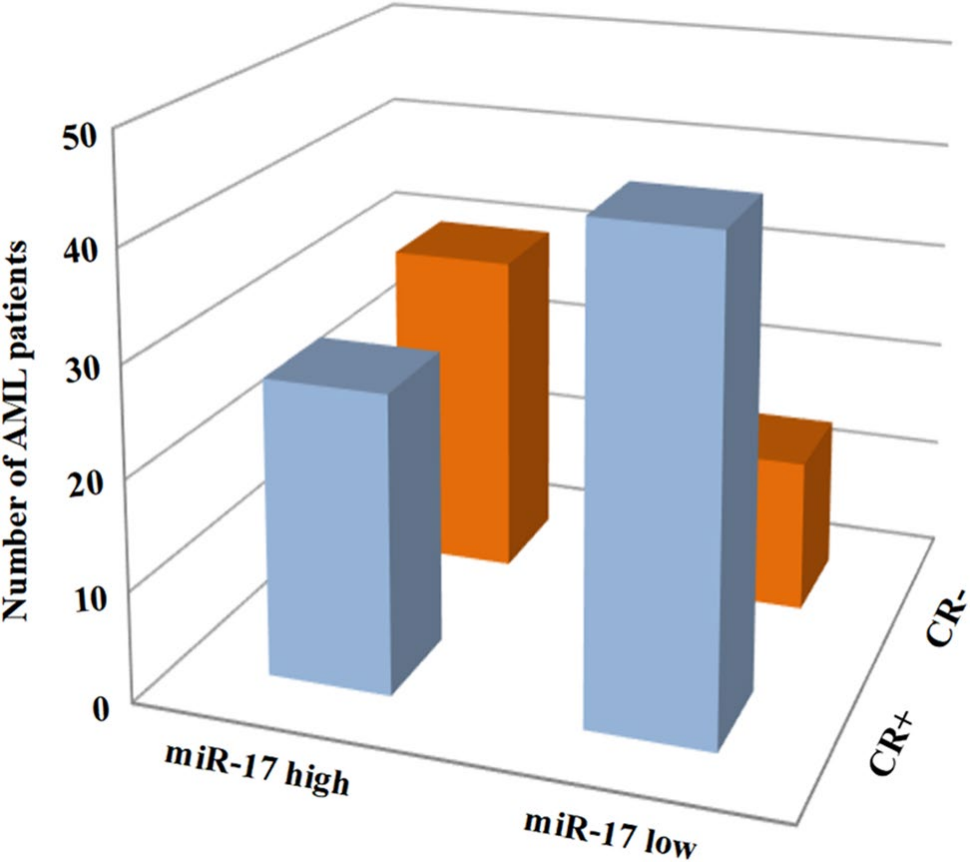
Correlation between miR-17 expression and CR state. The distributions of AML patients according to the miR-17 expression and CR state. *CR* complete remission

**Fig. 3:**
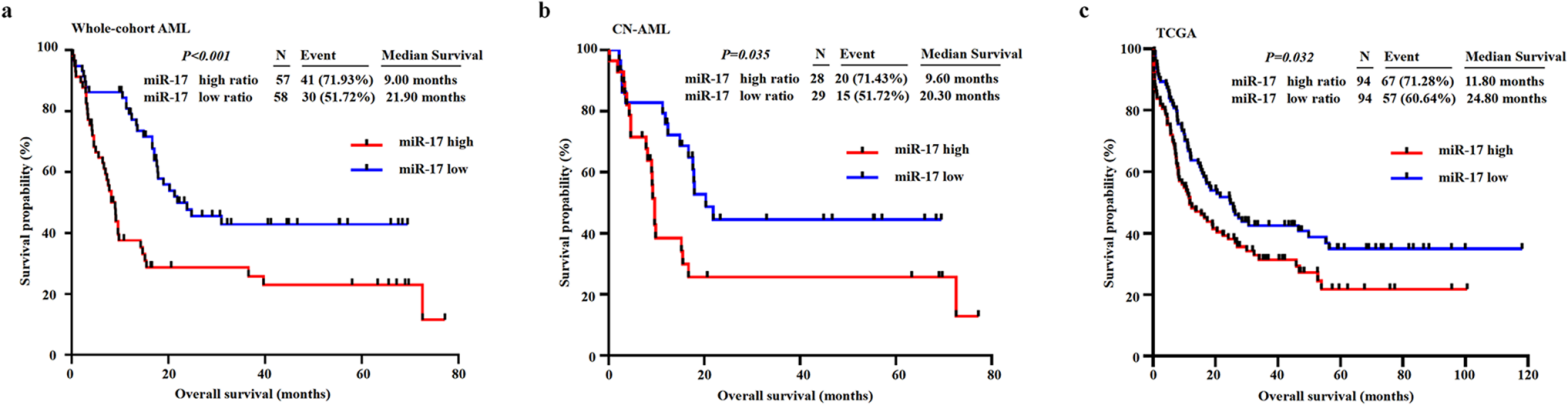
The impact of BM miR-17 expression on survival in AML patients. **a**, **b** Kaplan–Meier survival analysis based on high or low level of miR-17 expression in whole-cohort AML and CN-AML patients. **c** Kaplan–Meier survival analysis of the AML patients from TCGA dataset as a validation cohort

The AML patients with low miR-17 expression had higher chance for remission (*P* = 0.002; Table 1). 61.97% of patients with CR after induction therapy had lower miR-17 expression, and 68.18% of patients who did not achieve CR had higher miR-17 expression (Fig. 2). We further analyzed the prognostic value of miR-17 expression in AML patients. Kaplan–Meier analysis showed that the median OS time was shorter in the whole-cohort AML patients with high miR-17 expression than those with low miR-17 expression (*P* < 0.001; Fig. 3a). Similar result was obtained in CN-AML patients (*P* = 0.035; Fig. 3b). To further validate the adverse prognostic significance of miR-17 expression in AML, data from TCGA database was set as a validation cohort. A total of 188 AML patients were classified into two groups according to median level of miR-17 expression. Consistent with our results, the low expression of miR-17 was significantly correlated with longer OS (*P* = 0.032, Fig. 3c). Moreover, in the univariate and multivariate Cox analysis, miR-17 expression retained independent prognostic significance for OS even in the presence of other covariates, such as age and IDH1 mutation (Table 2).

**Table 2:** Univariate and multivariate analyses of prognostic factors for OS in AML patients

Variables	Univariate analysis		Multivariate analysis	
	HR (95% CI)	*P* value	HR (95% CI)	*P* value
miR-17 expression (high vs. low)	2.293 (1.414–3.716)	** < *0.001***	2.232 (1.348–3.698)	***0.002***
ELN risk (intermediate vs favorable) (adverse vs favorable)	2.166 (1.185–3.958) 4.287 (2.132–8.622)	** < *0.001*** ***0.012*** ** < *0.001***	1.457 (0.770–2.759) 1.608 (0.629–4.110)	0.463 0.248 0.321
Age (≥ 60 vs < 60)	2.861 (1.749–4.681)	** < *0.001***	2.346 (1.372–4.011)	***0.002***
TP53 (mutant vs. wild-type)	2.436 (1.106–5.368)	***0.027***	1.305 (0.454–3.748)	0.621
IDH1 (mutant vs. wild-type)	5.408 (2.111–13.855)	** < *0.001***	3.427 (1.078–10.897)	***0.037***
AML1-ETO (presence vs. absence)	0.289 (0.091–0.920)	***0.036***	0.516 (0.152–1.748)	0.288
WBC (≥ 20 vs. < 20 × 10^9^/L)	1.228 (0.764–1.972)	0.397		
BM blasts (%) (≥ 50 vs. < 50)	1.121 (0.648–1.940)	0.683		
CEBPA (double mutant vs. other)	0.468 (0.187–1.171)	0.105		
NPM1 (mutant vs. wild-type)	0.948 (0.470–1.909)	0.880		
FLT3-ITD (mutant vs. wild-type)	1.221 (0.624–2.387)	0.560		
TET2 (mutant vs. wild-type)	0.967 (0.480–1.947)	0.924		
RUNX1 (mutant vs. wild-type)	1.675 (0.764–3.672)	0.198		
ASXL1 (mutant vs. wild-type)	0.914 (0.287–2.909)	0.879		
IDH2 (mutant vs. wild-type)	0.828 (0.423–1.617)	0.580		
DNMT3A (mutant vs. wild-type)	1.602 (0.887–2.894)	0.118		

*HR* hazard ratio, *CI* confidence interval, *WBC* white blood cells, *BM*, bone marrow. Multivariate analysis included variables with *P* < 0.05 in univariate analysis

### miR-17 expression in the surveillance of AML

**Fig. 4:**
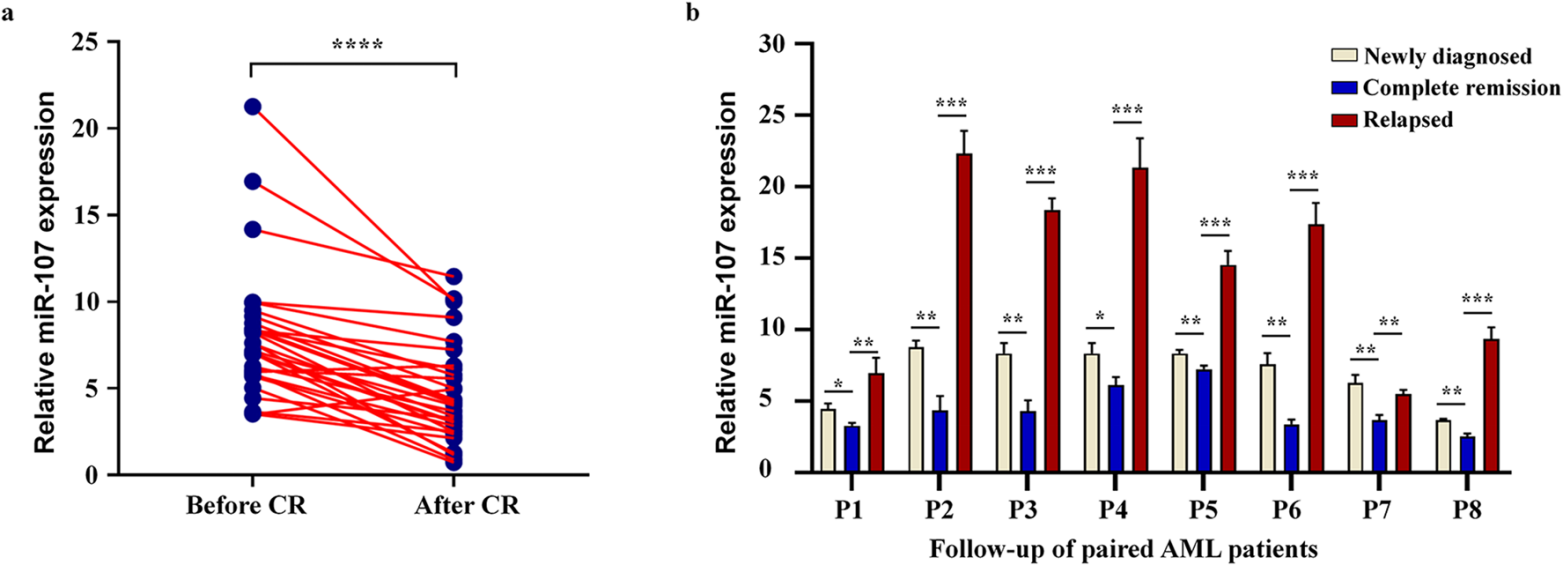
miR-17 expression in the follow-up of AML patients in different stages. **a** miR-17 expression levels in 31 AML patients achieving a CR before and after treatment. **b** Dynamic alteration of miR-17 expression in the 8 paired AML patients with different clinical stages. Data are shown as mean ± SD from three independent experiments

To monitor the dynamic alteration of miR-17 expression in the follow-up of AML patients, qPCR was conducted to assess miR-17 expression in 31 patients out of the 71 patients achieved CR. The expression of miR-17 significantly decreased after successful induction chemotherapy (*P* < 0.001, Fig. 4a). Moreover, the dynamic change of miR-17 expression was also detected in eight relapsed patients. The results indicated that miR-17 expression level was returned to primary level even higher when in relapse phase, which might predict disease recurrence in AML patients (Fig. 4b).

### Biological insights in AML of miR-17 profiles

**Fig. 5:**
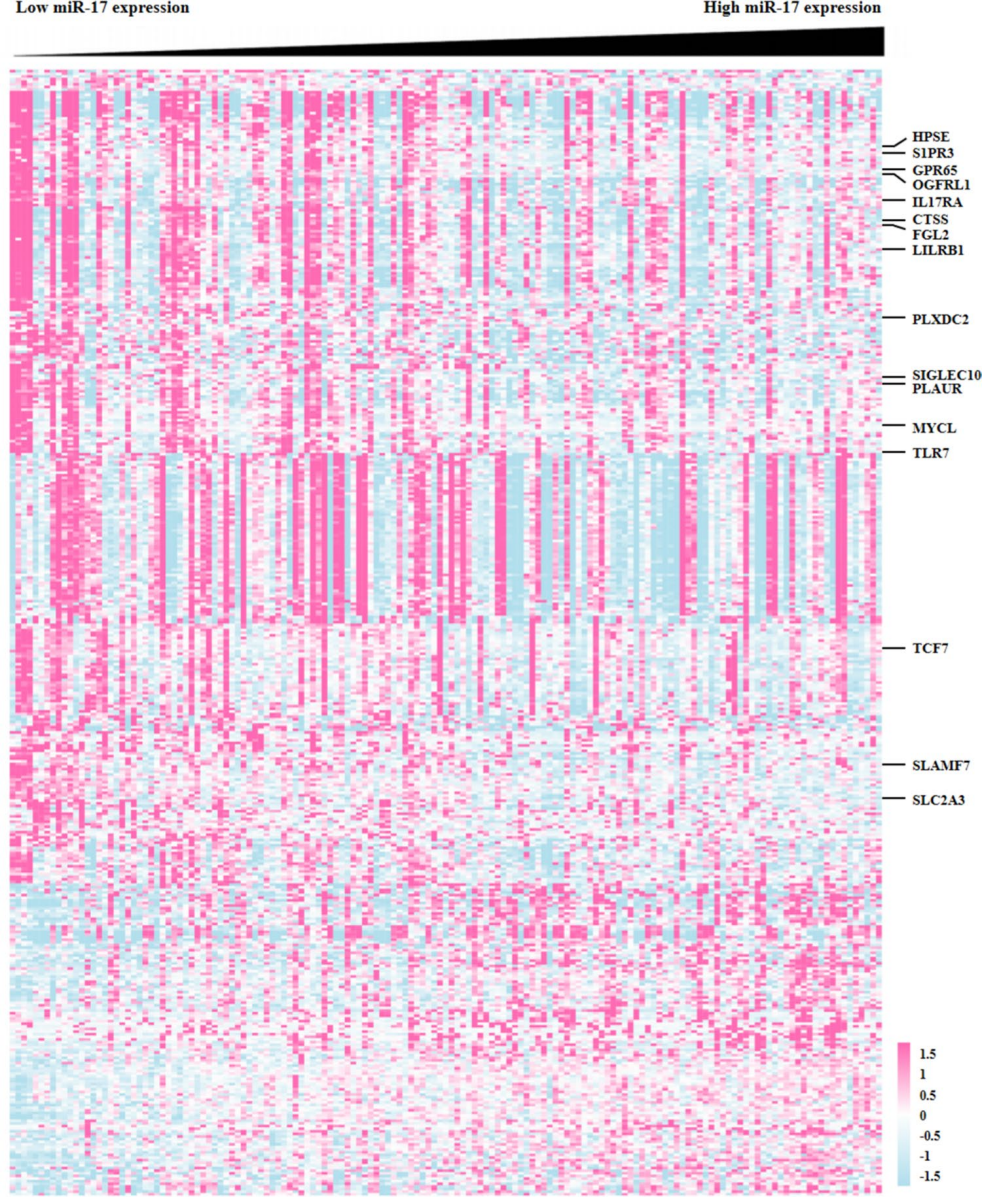
DEGs between AML patients with high and low miR-17 expression. The rows indicate the genes and the columns represent the patients. The patients were sorted in order from left to right by increasing levels of miR-17. Pink and blue represent expression levels above and below median gene expression (white), respectively. DEGs: differentially expressed genes

**Fig. 6:**
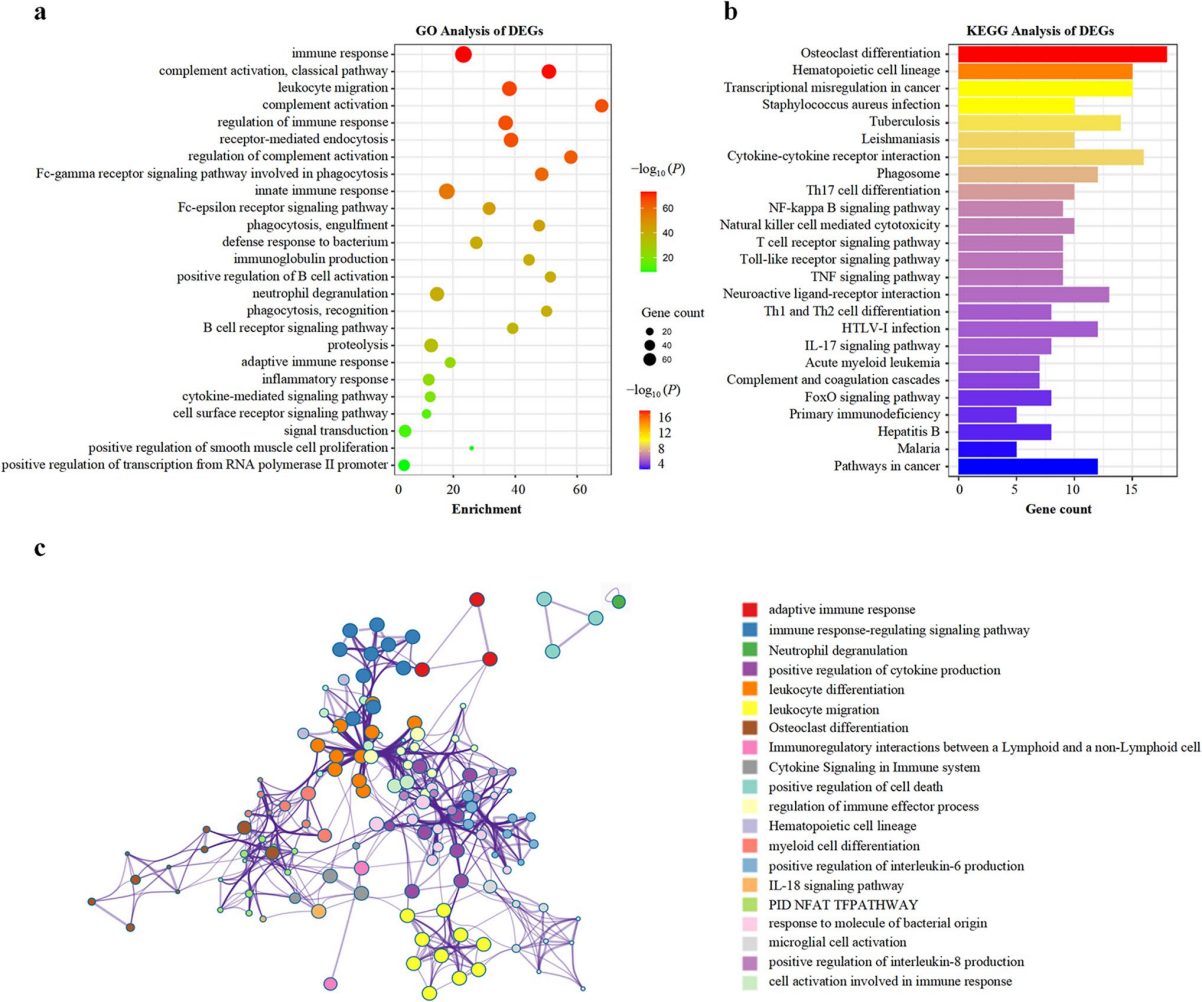
Functional enrichment analysis of DEGs. **a** GO and **b** KEGG analysis for hub genes by DAVID. **c** Enriched terms were colored by cluster ID, connected by edges and rendered as a network plot using the Cytoscape tool. *GO* Gene Ontology, *KEGG* Kyoto Encyclopedia of Genes and Genomes

**Fig. 7:**
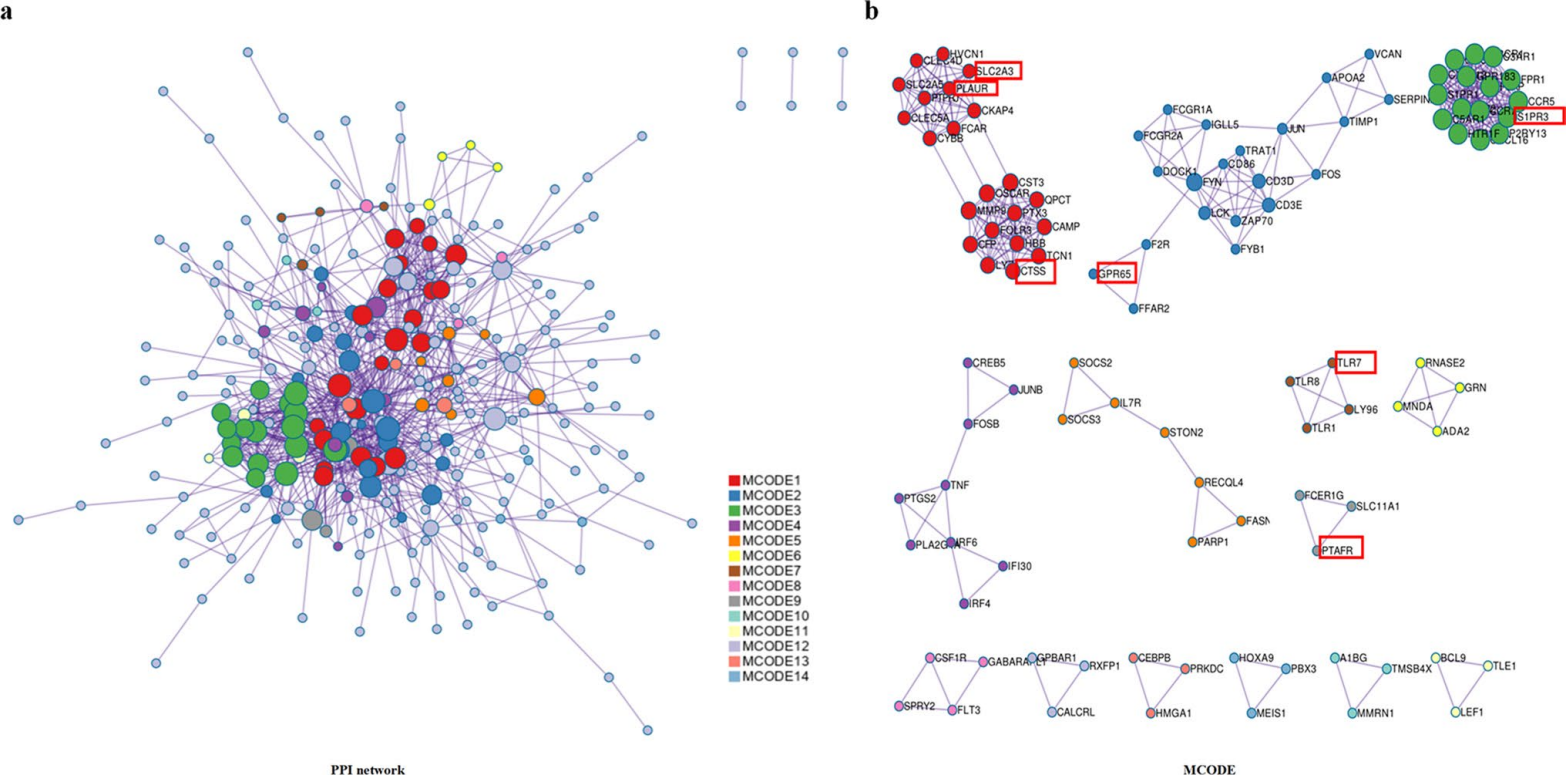
PPI network and MCODE components identified to be associated with the analysis of DEGs. **a** Overall PPI network of the DEGs. **b** Individual modules selected from the PPI network using the MCODE method. Each color represents each MCODE network. *PPI* protein–protein interaction; *MCODE* molecular complex detection

To further unveil the biological function of miR-17 in AML patients, we analyzed differential gene expression based on miR-17 level using the high throughput sequencing data of the AML cohort from TCGA database [8]. We found that 429 differentially expressed genes (DEGs) associated with miR-17 expression, among which 310 and 119 genes were negatively and positively correlated with miR-17 expression, respectively (Fig. 5). To screen for the target genes of miR-17, the online applications Targetscan, miRWalk and miRanda website tools were employed in silico analysis. Among the predicted target genes, miR-17 expression was inversely correlated with the expression of FGL2, PLAUR, SLC2A3, GPR65, CTSS, TLR7, S1PR3, OGFRL1, LILRB1, IL17RA, SIGLEC10, SLAMF7, PLXDC2, HPSE, TCF7 and MYCL. Gene Ontology (GO) analysis revealed that the DEGs associated with miR-17 expression were involved in immune response, complement activation, leukocyte migration, receptor-mediated endocytosis, phagocytosis, engulfment, positive regulation of B cell activation, immunoglobulin production and so on (Fig. 6a). Kyoto Encyclopedia of Genes and Genomes (KEGG) pathway analysis showed that the enriched pathways of the DEGs were involved in osteoclast differentiation, hematopoietic cell lineage, acute myeloid leukemia, transcriptional mis-regulation in cancer, cytokine-cytokine receptor interaction, pathways in cancer and so on (Fig. 6b). To further identify more proteins which belong to same pathway or have similar function, the 429 DEGs were clustered into 20 groups on the basis of their enrichment score and then rendered as a network plot (Fig. 6c). Furthermore, a protein–protein interaction (PPI) network of the DEGs was constructed and identified fourteen molecular complex detection (MCODE) modules (Fig. 7). Interestingly, seven targets of (PTAFR, TLR7, S1PR3, GPR65, CTSS, PLAUR and SLC2A3) were identified as hub genes of the MCODE modules. Therefore, the gene-expression profiling signature suggests that miR-17 plays important roles in regulating biological functions and signal pathways, which further confirms its clinical significance in AML patients.

## Discussion

In current study, we explored the prognostic value of miR-17 in de novo AML for the first time. The results showed that miR-17 frequently overexpressed in AML patients, which was significantly related to poor CR rate and shorter OS. Cases with high miR-17 levels had a lower frequency of CEBPA double mutation and less favorable risk according to ELN risk stratification. The follow-up of 31 patients achieved CR and 8 relapse patients revealed that miR-17 expression significantly decreased after successful induction chemotherapy and returned to primary level even higher when in relapse phase. Moreover, the gene expression profile of miR-17 involved in multiple biological functions and signal pathways. By bioinformatics analysis, we found FGL2, PLAUR, SLC2A3, GPR65, CTSS, TLR7, S1PR3, OGFRL1, LILRB1, IL17RA, SIGLEC10, SLAMF7, PLXDC2, HPSE, TCF7 and MYCL as direct targets of miR-17 among the DEGs. These results suggested that miR-17 might function as independent prognostic biomarker and predict disease recurrence for AML patients.

The miR-17∼92 cluster has been reported to be crucial for vertebrate development such as lymphocyte maturation [9], skeletal development [10], epithelial proliferation and branching [11]. Targeted deletion of miR-17∼92 in mice leads to early neonatal lethality with ventricular septal defects, lung hypoplasia, and B lymphopoiesis inhibition [12]. Accordingly, the miR-17∼92 cluster is involved in cardiovascular, neurodegenerative and immune diseases [13–15]. Other than the involvement in normal development described above, the majority of the previous studies have demonstrated the potential oncogenic role of miR-17∼92 cluster in various cancers. For instance, in retinoblastoma, miR-17∼92 acted as an *RB*-collaborating gene to promote retinoblastoma, in part by regulating p21Cip1 and p57Kip [16]. In colon cancer, miR-17 induced epithelial-mesenchymal transition and the formation of a stem cell-like population through the modulation of CYP7B1 expression [17]. In lung cancer, miR-17-5p was overexpressed and correlated with poor survival of patients [18]. In B-cell chronic lymphocytic leukemia (CLL), the miR-17∼92 cluster members were highly amplified, among which four (miR-17, miR-20a, miR-18a and miR-19b) and five (miR-17, miR-20a, miR-18a, miR-19a and miR-19b) members were significantly induced by CD154 and stromal cell culture, respectively [19]. Moussay et al*.* proved circulating miR-20a was a reliable classifier to distinguish CLL patients from healthy controls (AUC = 0.920) and associated well with the disease severity (*P* = 0.0242) [19]. The miR-17/92 cluster was also found overexpressed in B-cell Lymphomas, and the individual members miR-19a/19b were required and sufficient for the B-cell lymphoma tumorigenesis [20, 21].The study performed by Meenhuis A found miR-17/20 in combination with two other miRNAs promoted expansion and replating capacity of myeloid progenitors by targeting sequestosome 1–regulated pathways [22]. In another study, Li et al*.* showed miR-17/20 was particularly amplified in MLL-rearrangement AML patients [23]. However, there is some evidence that miR-17 also possesses antitumor properties. Aberrant low expression of miR-17, consistent with the high frequency loss of heterozygosity and deletions at 13q31.3, has been reported in several types of cancers [24, 25]. For example, in prostate cancer, miR-17 has been demonstrated to attenuate androgen receptor signaling and cell growth by targeting proto-oncogenic transcriptional activator PCAF [26]. In oral squamous cell carcinoma, miR-17 functioned as a tumor suppressor via regulating KPNA2/PI3K/AKT axis [27].

Here, we reported that miR-17 predicted poor prognosis and added to the prognostic value of various previously identified molecular indicators in AML, such as TP53, CEBPA, NPM1, FLT3-ITD and so on. In our study, APL patients were not enrolled, considering that it has shifted to a highly curable AML subtype with its own typically different genetic characteristics, risk stratification and target-therapy. The role of miR-17 as prognostic factor was not restricted to CN-AML but proved in a highly mixed population of AML including patients with old age and cytogenetic abnormalities. There was significant difference only in CEBPA double mutation between the high and low miR-17 groups. Though the regulation of CEBPA expression by miR-17 had been reported in several studies [28, 29], how miR-17 influences CEBPA double mutation is not fully illuminated. In line with our results, previous study found no significant association between miR-17 and NPM1 or FLT3 mutation status in AML patients [30]. In addition, there was evidence showing that miR-17 ~ 92 cluster contributed to the MEIS1/HOXA9-mediated transformation of MLL leukemia [31]. MLL is involved in over 100 different recurrent rearrangements, of which greater than 70 translocation partner genes (TPGs) have now been identified [32, 33]. Despite the vast number of partner genes, only nine TPGs (AF4, AF9, ENL, AF10, AF6, ELL, AF1P, AF17 and SEPT6) seem to be predominantly recombined to MLL. In our study, we detected MLL-partial tandem duplications (MLL-PTD) and eleven MLL-related fusion (MLL-AF9, MLL-AF4, MLL-ENL, MLL-AF10, MLL-SEPT6, MLL-ELL, MLL-AF17, MLL-AF1q, MLL-AF1p, MLL-AF6, MLL-AFX). In accordance with previous reports [34, 35], two of the four (50%) cases with MLL-related abnormalities were classified as M5 category indicating the commonly association with monocytic leukemias. The four MLL-related aberrations (including two MLL-PTD mutations and two MLL-related fusions) were exclusively found in high miR-17 group but unfortunately outside the significance level. The presence of distinct MLL lesions in AML is widely considered as an independent dismal prognostic factor despite improved regimen options like allogeneic hematopoietic stem cell transplantation appealing for novel effective regimens [36, 37]. Interestingly, the colony forming ability of MLL-fusion containing cells could be dramatically abolished in response to treatment with antagomir-17 [31]. Whether, therapy targeting miR-17 can benefit AML patients especially with the MLL-related aberrations or M5 subtype needs further study.

To derive biological insights into the oncogenic mechanism of miR-17 in AML, gene-expression profiling signature characteristic of miR-17 was analyzed. Bioinformatics data showed that those miR-17-associated genes were involved in hematopoietic cell lineage, transcriptional mis-regulation and pathways in AML. We found a negative correlation between miR-17 expression with sixteen miR-17-targeted genes among the DEGs. Notably, there were seven targets of miR-17 (PTAFR, TLR7, S1PR3, GPR65, CTSS, PLAUR and SLC2A3) identified as hub genes in the MCODE modules. Among them, PTAFR is a platelet-activating factor and involved in a wide range of human functions. The membrane expression of PTAFR, resulted from cell maturation and differentiation, was a marker of mature cells and rarely observed in AML blasts [38]. Fiedler ERC et al. have proved that PAFR could sensitize CML cells to the dasatinib treatment after binging with PAF [39]. Another previous study revealed PTAFR as a promising target for tumor repopulation induced by radiotherapy in solid tumor [40]. TLR7, namely Toll‐like receptor 7, was enriched into Toll-like receptor signaling pathway, inflammatory response and innate immune response. Ge F et al. have demonstrated TLR7 as a novel DNA methylation prognostic signature for AML patients who might benefit from TLR7‐based immunosuppressive therapy [41]. S1PR3, as an inflammation-activated S1P receptor, not only governed the myeloid fate in normal hematopoiesis via the TNFα–NF-κB axis, but also predicted prognosis in human AML [42]. GPR65, a member of purine receptor family, has been reported to suppress hematopoiesis by establishing repressive chromatin, repressing Gata2 transcription and thus inhibiting GATA-2-GPCR circuit [43]. CTSS, as lysosomal globular proteases, participated in various biological processes including immune response, neutrophil degranulation, proteolysis and so on. As summarized in a systematic review, the prognostic value of CTSS remains controversial in leukemia [44]. PLAUR, also known as CD87, has shown poor survival benefits in leukemias, with the potential to be used as a target for fusion protein therapy of PLAUR-expressing AML [45, 46]. SLC2A3, a regulator of early embryonic development, contributes to glucose transport and participates in multiple pathways [47]. Available evidence proved that low expression of SLC2A3 predicted poor response of demethylation and vitamin C, which had a worse effect on OS in AML [48]. Taken together, the gene-expression profiling associated with miR-17 might provide mechanistic insights into the clinical prognostic significance of miR-17 in AML.

In conclusion, our study revealed that miR-17 was upregulated in de novo AML patients, and its high expression pointed to dismal clinical outcome and disease recurrence. MiR-17 expression might serve as a novel independent prognostic biomarker for AML patients. Further studies are required to explore the biological roles of miR-17 and its target genes in AML.
